# Symbiosis of a lytic bacteriophage and *Yersinia pestis* and characteristics of plague in *Marmota himalayana*

**DOI:** 10.1128/aem.00995-24

**Published:** 2024-07-18

**Authors:** Dongyue Lyu, Qun Duan, Ran Duan, Shuai Qin, Xiaojin Zheng, Xinmin Lu, Asaiti Bukai, Peng Zhang, Haonan Han, Zhaokai He, Hanyu Sha, Di Wu, Meng Xiao, Huaiqi Jing, Xin Wang

**Affiliations:** 1 National Institute for Communicable Disease Control and Prevention, Chinese Center for Disease Control and Prevention, Beijing, China; 2 Akesai Kazakh Autonomous County Center for Disease Control and Prevention, Jiuquan, Gansu, China; Centers for Disease Control and Prevention, Atlanta, Georiga, USA

**Keywords:** *Yersinia pestis*, bacteriophage, symbiosis

## Abstract

Surveillance for animal plague was conducted in the *Marmota himalayana* plague focus of the Qinghai–Tibet Plateau from 2020 to 2023. A 22.89% positive rate of serum F1 antibody was detected in live-caught marmots, alongside a 43.40% incidence of *Yersinia pestis* isolation from marmot carcasses. Marmot carcasses infected with plague exhibited a significantly higher spleen-somatic index (*P* < 0.05). Twenty-one *Y. pestis-*specific phages were isolated, among which one *Y. pestis* lytic phage (AKS2022HT87GU_phi) was isolated from the bone marrow of a marmot carcass (no. AKS2022HT87) and was found to be symbiotic with *Y. pestis*. Microscopy revealed the coexistence of lysed and non-lysed colonies of *Y. pestis* AKS2022HT87. Genome-wide analysis showed that certain strains of the *Y. pestis* AKS2022HT87 carried phage DNA fragments consistent with phage AKS2022HT87GU_phi. The rare symbiotic relationship between a lytic phage and *Y. pestis* observed *in vitro* was highlighted in this study, laying the basis for further exploring the relationship between *Y. pestis* and its bacteriophages.

**IMPORTANCE:**

Bacteriophages and host bacteria commonly coexist *in vivo* or in soil environments through complex and interdependent microbial interactions. However, recapitulating this symbiotic state remains challenging *in vitro* due to limited medium nutrients. In this work, the natural symbiosis between *Yersinia pestis* and specific phages has been discovered in a *Marmota himalayana* specimen. Epidemiological analysis presented the characteristics of the *Y. pestis* and specific phages in the area with a strong plague epidemic. Crucially, comparative genomics has been conducted to analyze the genetic changes in both the *Y. pestis* and phages over different periods, revealing the dynamic and evolving nature of their symbiosis. These are the critical steps to study the mechanism of the symbiosis.

## INTRODUCTION

Bacteriophages are a kind of virus that can infect bacteria. Since the discovery of bacteriophages in the early 20th century, *Yersinia pestis-*specific phages have been reported in succession (1–3). *Y. pestis* widely exist in nature and traces of *Y. pestis* bacteriophages can almost always be found in the same locations (4). *Y. pestis* phages are capable of lysing *Y. pestis* and have high specificity, so have been widely used in the identification of *Y. pestis* and the diagnosis of plague, and attempts have been made to use them for the treatment of plague (5–7). In addition, these bacteriophages play a certain role in the evolution of *Y. pestis* (8). Different *Y. pestis* phages differ in antigenic properties, morphology, virulence, genome structure, and specificity for *Y. pestis* (4). For example, bacteriophage ΦA1122 is specific and efficient for the lysis of *Y. pestis* at 20°C but does not affect *Yersinia pseudotuberculosis* (5). In contrast, YpfΦ phage can infect various pathogenic species of the genus *Yersinia* as well as *Escherichia coli* (9). Genome sequences are available for many *Y. pestis* bacteriophages, including *Podoviridae* bacteriophages Yep-phi, Berlin, and Yepe2 and the *Myoviridae* bacteriophages L-413 and PY10 (10–12).

*Y. pestis* and its phages from different foci usually have distinct characteristics, but the antagonistic relationship between them is consistent with other phages and hosts in nature (13). The interaction of phages with the bacterial surface (adsorption) is the first step in the infection process and involves the recognition and binding of phage receptor-binding protein to one or more cell-envelope constituents, ultimately leading to ejection of phage DNA from the capsid (14). The investigation of plague in different natural plague foci may help identify the relationship between different species of *Y. pestis* and its specific phage.

The *Marmota himalayana* plague focus of the Qinghai–Tibet Plateau is the largest and most active focus in China (15). A previous plague outbreak among *M. himalayana* families posed a potential hazard to human health (16). The rapid development of tourism and economic trade, especially the existence of stripping marmots or skin trafficking, has led to cases of human plague and facilitated the long-distance spread of marmot plague (17, 18). In 2019, plague patients from Inner Mongolia were transferred to Beijing, and two cases were sent to Ningxia in 2021 (19, 20). Compared with the low virulence of *Y. pestis* in house rats in China, the virulence of *Y. pestis* in marmots is relatively higher (21). Moreover, in recent years, no human plague case from the *M. himalayana* plague focus survived; thus, if this plague spread over a long distance, the consequences would be unimaginable (15, 17).

To date, there are few reports of *Y. pestis* phage naturally existing in host animals (2). Furthermore, the interaction between *Y. pestis* and *Y. pestis* phage in host animals has yet to be fully elucidated and has become the focus of our research. *Y. pestis* phage vB_YpP-YepMm was first isolated from the bone marrow of *M. himalayana* in 2020 (2). In recent years, we have continuously monitored plague in the *M. himalayana* plague focus of the Qinghai–Tibet Plateau, observed pathological changes in marmots, identified strains of *Y. pestis*, and studied the *Y. pestis* bacteriophage. This paper describes the symbiosis of a lytic bacteriophage and *Y. pestis* and associated pathological changes in *M. himalayana*.

## RESULTS

### Prevalence of plague among marmots in the plague focus of the Altun Mountains

**Fig 1:**
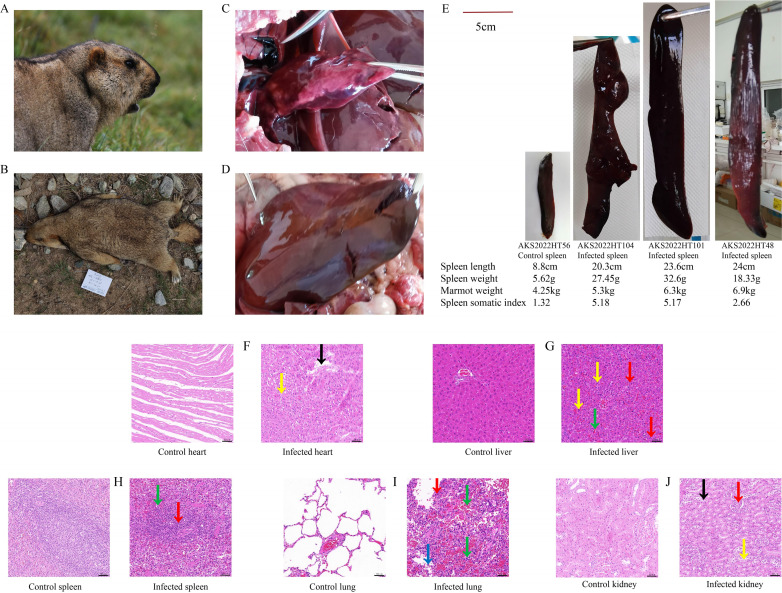
Gross lesions and histopathology of the organs of *M. himalayana* infected with *Y. pestis*. Images of: (A) live-caught marmot; (B) marmot carcass; (C–E) gross lesions; and (F–J) histopathology of various organs. Gross lesions show: (C) pulmonary nodule; (D) hepatosplenomegaly and hyperemia; and (E) control spleen and infected spleen. Histopathology was performed on uninfected (control) and infected samples for each organ: (F) heart (the black arrow points to the broken and dissolved myocardial fiber, the yellow arrow points to the cardiomyocytes with steatosis); (G) liver (the red arrows point to degenerated and necrotic hepatocytes, the green arrow points to congested and dilated hepatic sinusoid, the yellow arrows point to inflammatory cells); (H) spleen (the green arrow points to the spleen nodule, the red arrow points to the necrotic lymphocytes); (I) lung (the red arrow points to the proliferating alveolar epithelial cells, the green arrows point to the red blood cells in alveoli, the blue arrow points to the macrophage infiltration in alveoli); and (J) kidney (the red arrow points to glomerular lobulation, the black arrow points to the edematous renal tubular epithelial cell, the yellow arrow points to the renal tubular epithelial cell brush border).

*M. himalayana* is the main host animal in the *M. himalayana* plague focus. For each year from 2020 to 2023, the number of live-caught marmots in the plague focus of the Altun Mountains was 140, 160, 152, and 150, respectively (Fig. 1A). The seroprevalence of F1 antibodies among these marmots was 23.81% (30/126), 28.19% (42/149), 17.57% (26/148), and 22.07% (32/145), respectively. In addition, 48, 60, 107, and 50 marmot carcasses (referred to marmots that were found dead) were collected each year from 2020 to 2023, respectively (Fig. 1B), and the rates of *Y. pestis* isolation from these marmots were 35.42% (17/48), 51.67% (31/60), 51.40% (55/107), and 24.00% (12/50), respectively.

A total of 21 *Y. pestis-*specific phages were isolated, all of which were from marmot carcasses. Among them, 1 in 2020, 4 in 2021, 15 in 2022, and 1 in 2023 were isolated from bone marrow, heart, liver, spleen, lung, and kidney. Importantly, all phages can be isolated from bone marrow, and the positive rate of *Y. pestis* among these marmots with bacteriophages was 95.24% (20/21) (Table 1).

**TABLE 1:** Bacteriophage isolation from 2020 to 2023

Year	Bacteriophage count	Marmot number	Bacteriophage isolation site	Culture results for *Yersinia pestis^a^*
2020	1	dcw-bs-007	Bone	+
2021	4	AKS2021HT52	Bone	+
		AKS2021HT55	Bone, lung	+
		AKS2021HT58	Bone	+
		AKS2021HT71	Bone	+
2022	15	AKS2022HT 31	Bone	−
		AKS2022HT54	Bone, heart, spleen, lung, kidney	+
		AKS2022HT72	Bone	+
		AKS2022HT73	Bone, heart, liver, spleen	+
		AKS2022HT74	Bone, heart, liver, lung, kidney	+
		AKS2022HT75	Bone	+
		AKS2022HT77	Bone, heart, liver, spleen, lung, kidney	+
		AKS2022HT78	Bone	+
		AKS2022HT80	Bone	+
		AKS2022HT85	Bone	+
		AKS2022HT87^*b*^	Bone	+
		AKS2022HT88	Bone	+
		AKS2022HT 89	Bone	+
		AKS2022HT100	Bone	+
		AKS2022HT104	Bone, liver, spleen, kidney	+
2023	1	AKS2023HT30	Bone	+

^*a*^ *+: Y. pestis* was isolated; −: *Y. pestis* was not isolated.

^*b*^ The marmot in this study.

### Gross lesions and histopathology of the organs of marmots infected with *Y. pestis*

Compared with live-caught marmots, marmot carcasses infected with *Y. pestis* often had nasal bleeding, subcutaneous hyperemia, gastric serosa and mesentery hyperemia, heart enlargement, pulmonary hemorrhage, pulmonary nodules (Fig. 1C), hepatosplenomegaly, and hyperemia (Fig. 1D). Splenic lesions the most common lesions (Fig. 1E).

The histopathological changes in marmots infected with *Y. pestis* are mainly shown in the following aspects:

Heart: parts of the myocardial fibers were broken and dissolved (black arrow); mild steatosis in local cardiomyocytes; and regular round vacuoles were visible in cells (yellow arrow) (Fig. 1F).

Liver: some hepatocytes were degenerated and necrotic, with the nucleus exhibiting fragmentation, pyknosis, and deep staining (red arrow); mild hyperemia and dilatation could be seen in part of the hepatic sinusoid (green arrow); and a small number of inflammatory cells were observed in the tissue (yellow arrow) (Fig. 1G).

Spleen: the shape of the splenic nodule in the internal visual field was scattered, and the boundary between the spleen nodule and the red pulp was blurred (green arrow); a large number of lymphocytes in the white pulp were necrotic, and the nucleus of the lymphocytes was pyknotic and deeply stained (red arrow) (Fig. 1H).

Lung: local alveolar epithelial cells became hyperplasic and the alveolar septum exhibited slight thickening (red arrow); massive hemorrhage of tissue and a large number of red blood cells in some alveoli (green arrow); and macrophage infiltration in the partial alveoli (blue arrow) (Fig. 1I).

Kidney: part of the glomerular lobulation was obvious (red arrow); a large area of renal tubular epithelial cell edema, cell swelling, and cytoplasmic light staining (black arrow); and partial renal tubules were injured and the renal tubular epithelial cell brush margin had been shed (yellow arrow) (Fig. 1J).

### Comparison of the spleen-somatic index in different groups and months

**Fig 2:**
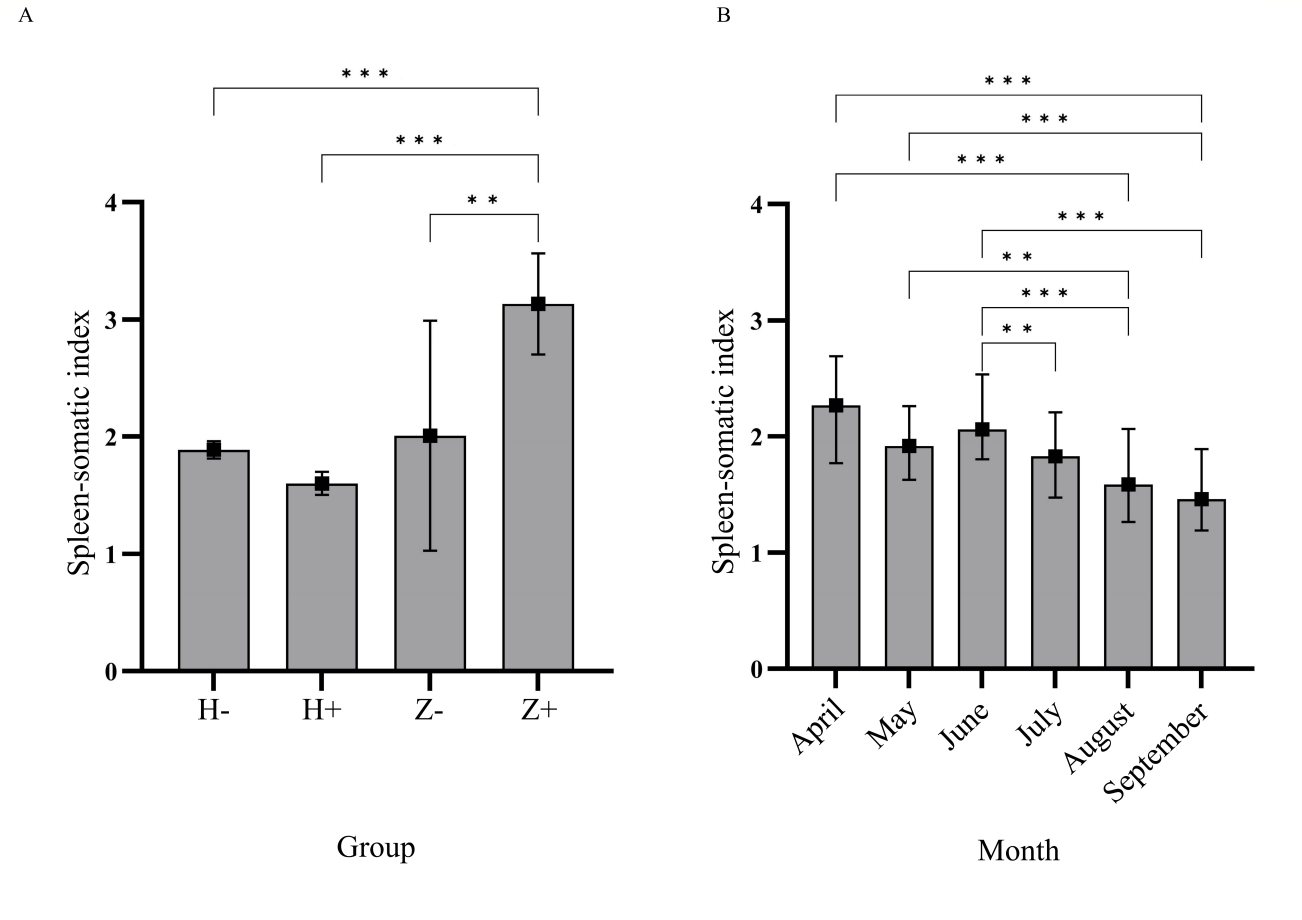
Spleen-somatic index comparison of marmots in different groups and months. (A) Different groups (H−: live-caught marmots with negative F1 antibody; H+: live-caught marmots with positive F1 antibody; Z−: marmot carcasses from which no *Y. pestis* were isolated; Z+: marmot carcasses from which no *Y. pestis* were isolated). (B) Different months. **P < 0.01; ***P < 0.001.

From 2020 to 2023, the spleen-somatic indexes of 567 marmots were obtained, of which 22 live-caught marmots did not have their blood collected. For comparison, live-caught marmots were divided into healthy marmots (H−) and previously infected marmots (H+) according to serological results for the *Y. pestis* F1 antibody. Marmot carcasses were divided into uninfected marmots (Z−) and infected marmots (Z+) according to the isolation of *Y. pestis*. Thus, 545 marmots were included in the final analysis and were grouped as 345 H−, 114 H+, 24 Z−, and 62 Z+, and the median and interquartile range (IQR) of the spleen-somatic index in the four groups were 1.78 (1.46–2.14), 1.62 (1.37–1.93), 1.71 (0.94–2.44), and 2.63 (2.20–3.74), respectively. The results of the Kruskal-Wallis test showed that the spleen-somatic index of the Z+ group was higher than that of the H− group (*H* = 167.88, *P* < 0.01), the H+ group (*H* = −216.16, *P* < 0.01) and the Z− group (*H* = −188.56, *P* < 0.01), respectively (Fig. 2A).

In addition, the spleen-somatic indexes of live-caught marmots were compared according to the sample collection month. The spleen-somatic index of live marmots captured in August and September was lower than that from April to June, while the average geometric titer of the F1 antibody was higher in August and September (Fig. 2B).

### Symbiosis of *Y. pestis* AKS2022HT87 and bacteriophage AKS2022HT87GU_phi

**Fig 3:**
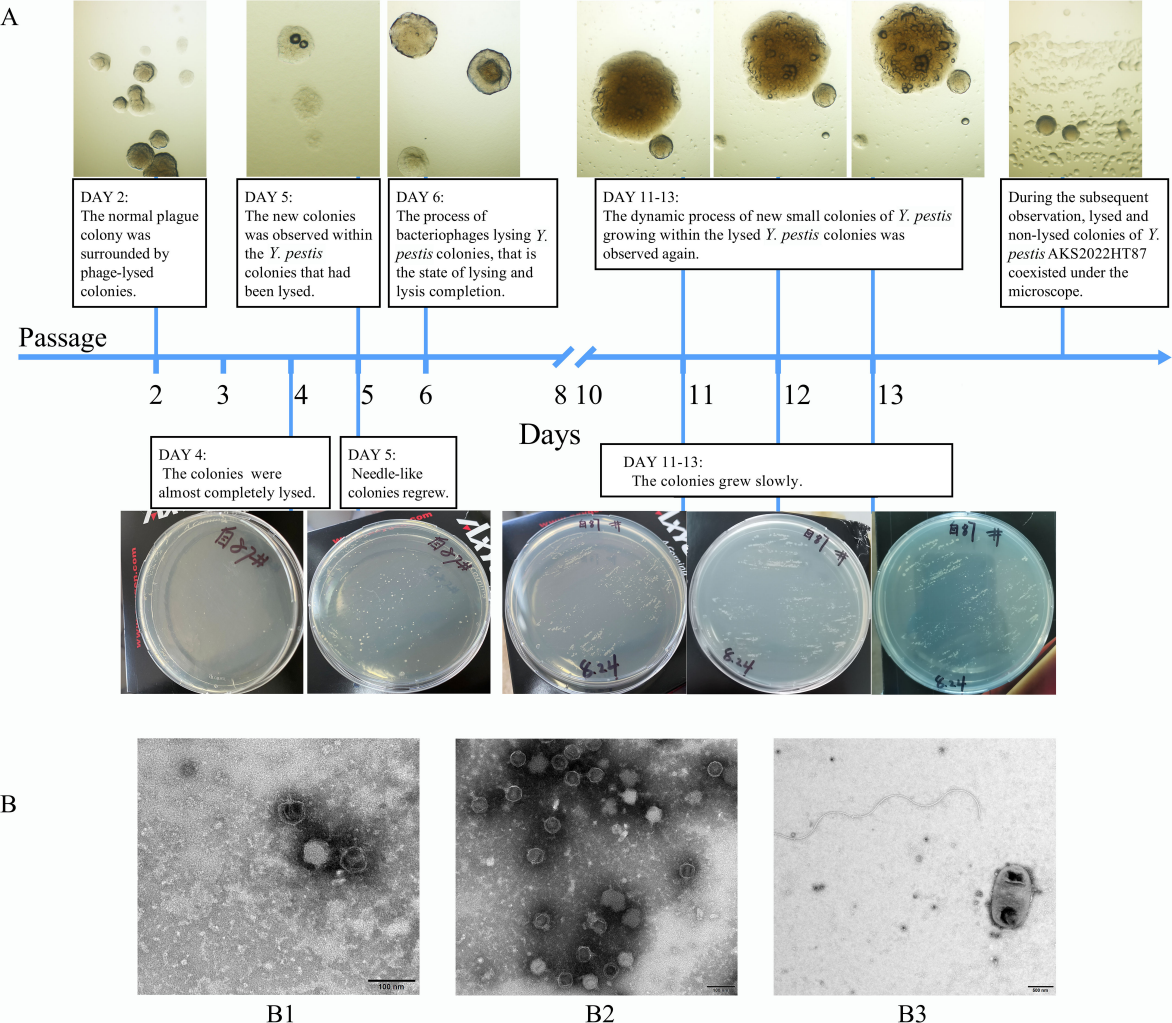
Strain growth of AKS2022HT87 and electron microscopy of phage AKS2022HT87GU_phi. (A) Under the microscope (1,000 magnification) and visual observation; (B) electron microscopy of phage (B1 and B2. the phage AKS2022HT87GU_phi was tadpole-shaped, with an iso-spaced hexagonal head and short, noncontractile, conical tails; B3. phages could be seen around the bacteria).

*Y.pestis* AKS2022HT87 isolated from the bone marrow of *M. himalayana* AKS2022HT87 grew very slowly at 28°C; the needle-like colonies could not be observed by the naked eye until 72 h on *Brucella* medium. *Y. pestis* AKS2022HT87 colonies were repeatedly lysed and regenerated. Lysed and non-lysed colonies of *Y. pestis* AKS2022HT87 coexisted under microscopy, indicating that there was a symbiosis of *Y. pestis* AKS2022HT87 and bacteriophage. The details are as follows (Fig. 3A):

1. The bone marrow sample of marmot carcasses AKS2022HT87 was inoculated onto *Brucella* medium and then passaged because of the small number of colonies.
2. On the second day of passage, the normal plague colony was surrounded by phage-lysed colonies microscopically.
3. On day 4, the colonies on the medium were almost completely lysed.
4. On day 5, needle-like colonies regrew on the medium, and the microscopic formation of new colonies was observed within the *Y. pestis* colonies that had been lysed by bacteriophage.
5. On day 6, the process of bacteriophages lysing *Y. pestis* colonies, that is the state of lysing and lysis completion, was visible under the microscope.
6. On days 11–13, the colonies grew slowly on the medium, and the dynamic process of new small colonies of *Y. pestis* growing within the lysed *Y. pestis* colonies was again observed under the microscope.
7. During the subsequent observation, needle-like colonies were still visible on the medium, and lysed and non-lysed colonies of *Y. pestis* AKS2022HT87 coexisted under the microscope.

### Characterization and genome-wide analysis of phage AKS2022HT87GU_phi

Under electron microscopy, the phage AKS2022HT87GU_phi was tadpole-shaped, with an iso-spaced hexagonal head and short, noncontractile, conical tails (Fig. 3B1 and B2). Phages could also be seen around the bacteria (Fig. 3B3). According to morphological phage classification, the phage AKS2022HT87GU_phi is a tailed phage in the family *Podoviridae*.

**Fig 4:**
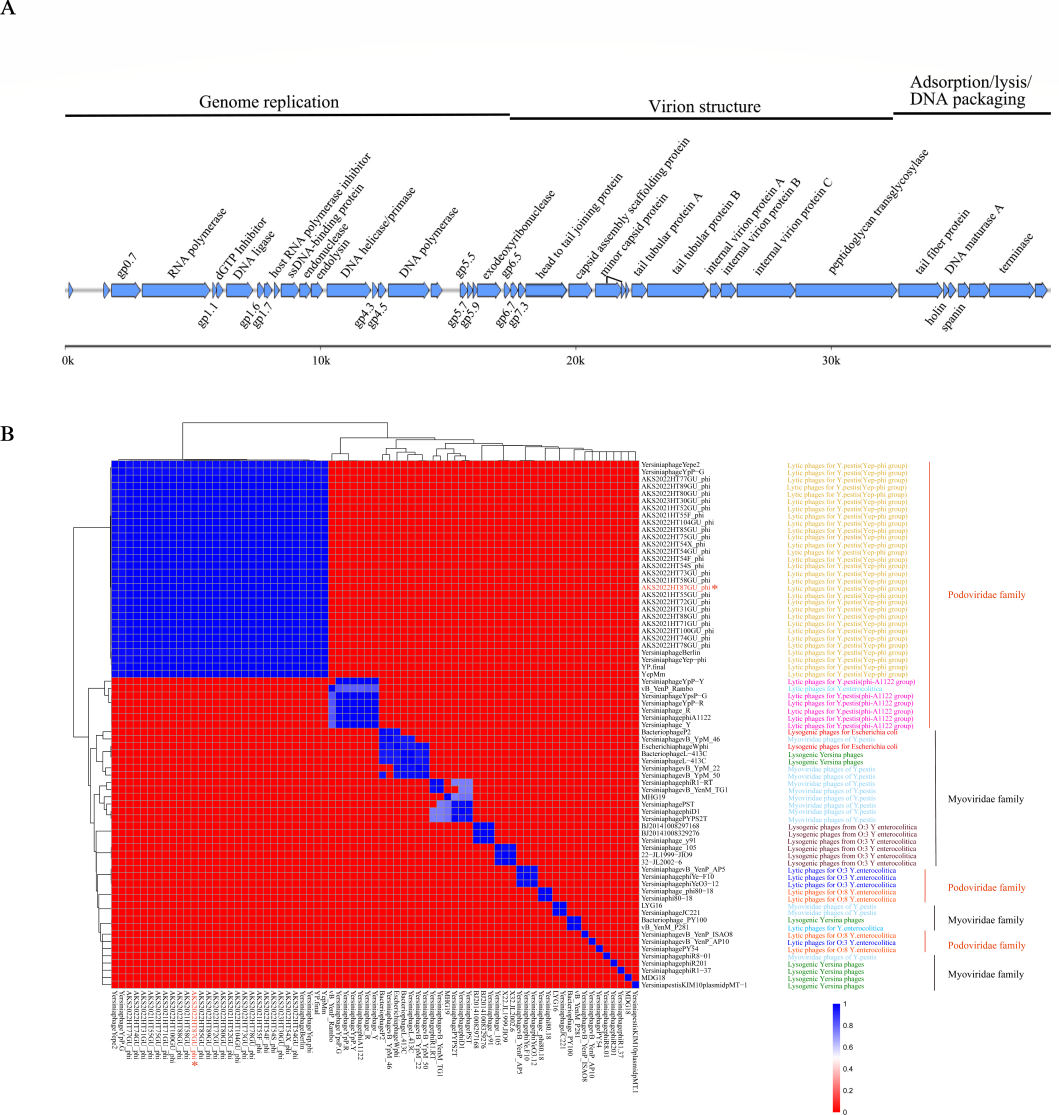
Phage genome structure (**A**) and average nucleotide identity matrix (**B**) of phages. * Indicates the bacteriophage (AKS2022HT87GU_phi) of *Yersinia pestis* in this study.

The genome length of the phage AKS2022HT87GU_phi is 38,649 bp (GenBank accession number OR671250). Based on the Swiss-Prot database, 43 hypothetical genes were predicted (Fig. 4A). Homologous retrieval analysis of the hypothetical genes revealed 37 genes with known functions, while the remaining genes failed to match the hypothetical proteins with known functions. The genome structure of the phage included specialized modules for gene regulation, genome replication, DNA restriction, DNA packaging, phage morphology, and bacteriolysis, which have been frequently observed in members of the family *Podoviridae*. None of the genes associated with lysogeny and bacteriophage virulence genes were found in the phage genome.

The phage phylogenetic tree was divided into two large branches (Fig. 4B). The genomes of plague lytic phages were highly similar. The DNA sequence of phage AKS2022HT87GU_phi was highly consistent with that of previously isolated lytic *Y. pestis* phages vB_YpP-YepMm (GenBank accession number MW767996, with sequence identity 99.99%), and it exhibited sequence identity of 98.26%, 97.68%, 96.63%, and 96.51% with the lytic plague phage Yep-4phi, Berlin, Yepe2, and YpP-G, respectively. These phages clustered together in a branch of the phylogenetic tree and had no homology with *Yersinia enterocolitica* phages and *Yersinia* lysogenic phages that formed another large branch of the tree.

### Difference and phylogenetic tree of *Y. pestis* AKS2022HT87

Two bacterial colonies—the normal-growing bacteria (strain A, GenBank accession numbers: CP155632, chromosome; CP155633, PMT1 plasmid; CP155634, pCD plasmid; and CP155635, pPCP plasmid) and the slow-growing bacteria (strain B, GenBank accession numbers: CP155628, chromosome and pPCP plasmid; CP155629, PMT1 plasmid; CP155630, phage DNA fragment; and CP155631, pCD plasmid)—from *Y. pestis* AKS2022HT87 were detected at different times, strain A could be lysed by phage, whereas strain B displays a needle-like appearance on the medium, with some regrowth occurring from within the colony (Fig. 3A). The whole genome of each of these two colonies was sequenced. The total length of chromosome sequences of strain A and strain B was 4,650,170 and 4,649,813 bp, and the GC content was 47.63% and 47.64%, respectively. Strain A carried three plasmids—pMT1, pPCP, and pCD—and strain B carried the same three plasmids plus a fourth “plasmid,” which was a 38,276 bp phage DNA fragment that was not integrated into the bacterial chromosome. The phage DNA fragment observed in strain B has a GC content of 47.3%, compared to 47.2% in phage AKS2022HT87GU_phi. This fragment demonstrated 100% coverage and 99.16% identity with the active phage AKS2022HT87GU_phi. Based on the Clusters of Orthologous Groups of proteins (COG) database, the chromosome of strain A predicted more functional proteins than that of strain B; these proteins included tRNA and RNase P protein components related to translation and ribosome structure, Membrane protein inserts related to cell membrane structure, Trans-2-enoyl-CoA reductase related to lipid transport and metabolic function, and so on. Comparison of the predicted proteins from Swiss-Prot databases showed almost no difference between the chromosomes of the two strains, while comparison by NR databases showed that strain B had more mobility genes such as transposase and integrase than strain A.

**Fig 5:**
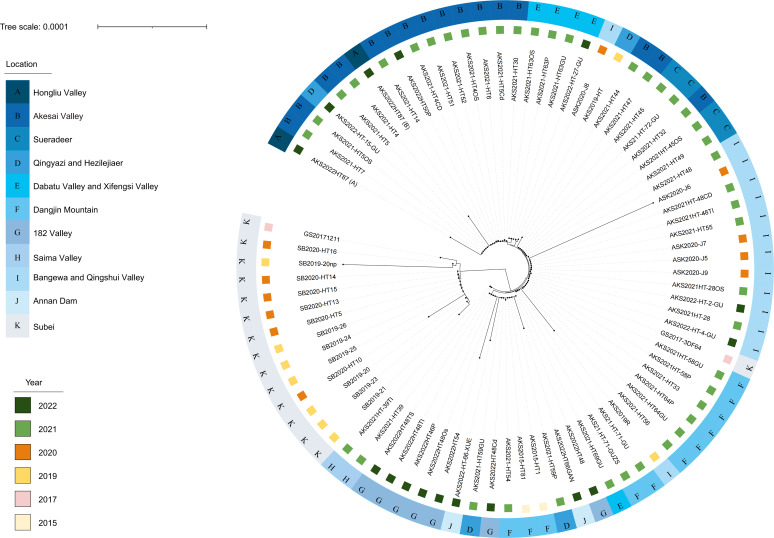
Phylogenetic tree based on core genes of 82 isolates of *Y. pestis* from the *M. himalayana* plague focus of the Qinghai–Tibet Plateau.

The two strains (strain A and strain B) of *Y. pestis* AKS2022HT87 were sequentially compared with 80 isolates of *Y. pestis* from the *M. himalayana* plague focus of the Qinghai–Tibet Plateau. Four of the isolates were from fleas, two were from plague cases, and the rest were from marmots. These 82 isolates tested positive for the *Pla* and *Caf1* genes by PCR and were identified as *Y. pestis* (identity was 100%). The phylogenetic tree based on 3,850 core genes showed that *Y. pestis* AKS2022HT87 was located on the same branch as outbreak strains isolated in 2021, and the geographical distance was consistent, that is, the closer the geographical distance, the closer the phylogenetic tree; however, there were differences in the evolutionary distance (Fig. 5).

## DISCUSSION

Plague remains endemic in African countries such as Madagascar and Congo (22–25), as well as in Mongolia and China (15, 26). The *M. himalayana* plague focus of the Qinghai–Tibet Plateau is the most widespread area of animal plague and the most serious threat to humans, often causing sporadic human plague cases with a high fatality rate (26). *Y. pestis* has been isolated in the *M. himalayana* plague focus every year since the first isolate was recovered in 1954. The strains derived from marmots possess strong virulence, with tens of *Y. pestis* being able to kill half of the exposed mice. In this study, animal plague surveillance in the *M. himalayana* plague focus in the Altun Mountains of the Qinghai–Tibet Plateau from 2020 to 2023 revealed that about 43.40% of marmot deaths were attributed to plague. It is anticipated that the mortality rate among plague-infected marmots is not low. The potential threat of animal plague still exists in this region, and that monitoring needs to be strengthened continuously. A healthy marmot has an average lifespan of 8–9 years and can live up to 12–15 years. They can be categorized as baby marmots, 1-year-olds, 2-year-olds, and adult marmots over 3 years old based on their sexual development. Marmots hibernate from September to October each year through to the end of March or mid-to-early April of the following year. F1 antibody titers of marmots before hibernation were generally high in protection, consistent with the higher antibody titers in our specimens collected in August and September. We observed multiple organ lesions in marmots infected with plague, with splenic hemorrhage and enlargement being the most common (27). *Y. pestis* is lymphophilic and can resist phagocytosis and killing by macrophages and polymorphonuclear leukocytes (28, 29). The spleen, which is the largest lymphoid organ in the body, is an important storage organ for macrophages and thus is the main target of *Y. pestis* invasion, resulting in a series of serious inflammatory reactions such as hemorrhage and enlargement (30). The spleen-somatic index was used to objectively reflect the degree of spleen enlargement and the immune status of marmots infected with plague in our study. The samples collected in August and September had a higher geometric mean titer of antibody, but a smaller spleen-somatic index, indicating that there was a protective F1 antibody in the marmots that may also affect the spleen weight. The pathological changes of the organs mainly manifested as bleeding, and the gross lesions of the organs were more serious than the histopathological changes, which may be because *M. himalayana* plague is a very rapid disease with a short course, causing toxic shock syndrome, and has not yet caused tissue lesions.

*Y. pestis* phages are bacterial viruses widely distributed in plague foci, such as in the infected animals, rodent hosts, and their habitats, and so on (4). We first isolated a *Y. pestis* phage from marmot bone marrow in 2020 (2), and subsequently have isolated many *Y. pestis* phages from *M. himalayana* carcasses in the following 3 years. As the most important source of phage isolation, bone marrow may be the “repository” of phage. One of the phages was isolated from the remains of bone marrow, without isolation of a *Y. pestis* strain. However, nucleic acid extracted from the bone marrow was positive for *Y. pestis-*specific genes. Considering the marmot carcasses may have been decaying for a long time in the environment, the bacteria may have died. However, the *Y. pestis* phage was still alive, which coincides with the view that the survivability of phage is stronger than that of bacteria in the environment (31). In addition, bacteriophage lysis toward bacteria may have played a role and cannot be excluded.

We discovered the “lysis-regeneration” phenomenon of bacteria and bacteriophages in this study. In addition to the common relationships between *Y. pestis* and bacteriophages, such as lytic and lysogenic interactions, there are carrier state and pseudolysogeny (32–34). A characteristic feature ascribed to the state described as the carrier state life cycle (CSLC) is that bacteriophages are constantly being generated within the culture at the expense of a sensitive cell population. When first isolated, strains exhibiting CSLC resemble lysogens in that they appear to be resistant to superinfection, but differ from most lysogens in that the bacteriophage nucleic acid does not appear to be integrated into the host genome (35). These characteristics parallel the scenario of phage and *Y. pestis* in this study. The carrier state has been observed in strictly lytic phages and temperate phages. For example, lytic group III bacteriophages, CP8 and CP30A, remain associated in equilibrium with their host bacteria *Campylobacter* (35). Temperate phages of the spBeta group use a small-molecule communication system to coordinate lysis–lysogeny decisions (36). In this study, the structure, whole-genome sequence, and phylogenetic tree of the phages showed that the gene structure of phage AKS2022HT87GU_phi is congruent with that of lytic phages Yep-phi, Berlin, Yepe2, and YpP-G (36). Therefore, phage AKS2022HT87GU_phi is a lytic phage with a similar status described as the CSLC. The persistence of phages through the destruction of susceptible *Y. pestis* during a single colony subculture coupled with the evidence for the presence of phage DNA in the culture support the idea that the lytic virulence life cycle of the phage has been interrupted.

The symbiosis of lysed and non-lysed colonies under microscopy demonstrates the constant evolutionary arms race between phage and *Y. pestis* (13, 37). Previous studies have shown that the coevolution of *E. coli* and T7, or the closely related phage T3, in chemostats rapidly reach stagnation, typically after only 1.5 cycles (bacteria-phage-bacteria mutations) (13). Therefore, antagonistic coevolution between bacteriophages and host bacteria is common and has been proven *in vivo* and the soil environment (38–40). Our study also indicates that *Y. pestis* and bacteriophage could be isolated simultaneously *in vivo*. However, antagonistic coevolution is rarely found *in vitro* owing to the limited nutrients of the culture medium; consequently, the symbiosis phenomenon captured in this study is a competitive state and is rare.

To explore the changes that occurred during symbiosis, we used strain A and strain B of *Y. pestis* AKS2022HT87 which were detected at different times. Strain B was found to carry a phage DNA fragment, whereas strain A did not. This difference in strains may be one reason for the symbiosis between *Y. pestis* and bacteriophages. Phages may infect sensitive bacteria but not tolerant bacteria. When bacteria are challenged by lytic infections, phage-resistant bacteria and phages that can overcome bacterial resistance both have selective advantages (41, 42). Therefore, these selective pressures could induce continuous mutual adaptation and evolve new resistance and anti-resistance pathways (43), resulting in high spatiotemporal diversity of bacterial species and bacteriophage genomes in nature (13). The symbiosis of *Y. pestis* and lytic phages also may be caused by concurrent mutation of the lytic and lysogenic cycles. In addition, phage AKS2022HT87GU_phi is a tailed phage, and most tailed phages infect *Y. pestis* by binding bacterial receptors with their tail proteins (12). Thus, the tail protein structure of phage AKS2022HT87GU_phi may be crucial to the symbiosis between specific lytic phages and *Y. pestis. Staphylococcus aureus* bacteriophages can develop unique proteins that bind and inactivate key bacterial proteins, which disrupts the main metabolic processes of bacteria and impedes vital cell growth processes, thereby slowing bacterial growth and redirecting bacterial metabolism toward the reproduction of phage offspring (44).

If the symbiosis of *Y. pestis* and a lytic phage *in vitro* similarly occurs in *M. himalayana*, then doubts will be raised about the therapeutic effect of *Y. pestis* bacteriophages. The phenomenon indicates that *Y. pestis* bacteriophages can reach a balance with *Y. pestis* during their interaction and coexist within marmots. Although some researchers have reported using *Y. pestis* phages to cure four plague cases, there is no case-control to confirm the true role of *Y. pestis* bacteriophages in the treatment of plague (4, 45, 46). We once determined *Y. pestis* phages from two pieces of bone marrow that were isolated from *Y. pestis*-infected marmots in the previous study and found that there were approximately 10^9^ plaque-forming units (PFUs) of bacteriophage particles per gram of bone marrow. Streaking this bone marrow on a solid medium mainly produced pure cultures of *Y. pestis* colonies (unpublished information). Some cultures were contaminated and produced a small number of miscellaneous bacteria, but *Y. pestis* remained predominant. Further research is required to fully elucidate the mechanisms and interactions between *Y. pestis* and its lytic bacteriophages that maintain their symbiotic relationship in nature.

## MATERIALS AND METHODS

### Sample sources

We conducted animal plague surveillance in the *M. himalayana* plague focus in the Altun Mountains of the Qinghai–Tibet Plateau from 2020 to 2023. Live-caught marmots were captured in historical animal plague epidemic areas during active surveillance. Marmot carcasses were predominantly found by herdsmen who reported to the Centers for Disease Control and Prevention (CDC), or by epidemiological investigators of the CDC during field investigation. Under the conditions of satisfying animal ethics, the live-caught marmots were euthanized the heart blood of them was drawn, and the serum was isolated. Euthanized live marmots (referred to as live-caught marmots) and marmot carcasses were weighed and dissected, and the heart, liver, spleen, lung, kidneys, and bone were collected. Gross lesions in the spleens of a live-caught marmot (no. AKS2022HT56) and three marmot carcasses (AKS2022HT48, AKS2022HT101, and AKS2022HT104) were observed. In addition, the collected organs were embedded in paraffin, sectioned, and stained with hematoxylin and eosin for histopathological analysis (47). For marmot remains that had rotted owing to the length of time since death or had been eaten by other animals, only the bones are collected.

live-caught marmots were divided into healthy marmots (H−) and previously infected marmots (H+) according to serological results for the *Y. pestis* F1 antibody. Marmot carcasses were divided into uninfected marmots (Z−) and infected marmots (Z+) according to the isolation of *Y. pestis*. The spleen-somatic index, calculated as spleen weight (g)/marmot weight (kg) (48), was compared in different marmot infection groups and sample collection months.

### *Y. pestis* isolation, identification, nucleic acid detection, and serological testing

Tissue samples were applied onto a Yersinia-selective agar (Oxoid) for *Y. pestis* isolation at 28°C for 48 h. Nucleic acids of the collected samples were extracted using a Blood & Tissue Kit (catalog no. 69506, Qiagen, Germany) and *Y. pestis*-specific genes (*pla* and *caf1*) were detected by PCR.

The colloidal gold-based immunochromatographic strip was developed for specific, sensitive, and rapid detection of *Y. pestis* F1 antibodies in multiple species (Beijing Jianaixi Biotechnology Co., Ltd., Beijing, China) and the indirect hemagglutination assay was performed for verification (Qinghai Province Endemic Disease Prevention and Control Institute, Xining, Qinghai Province, China). F1 antigen inhibition controls, negative controls, and positive controls were established. An antibody titer ≥1:16 was identified as positive.

### Bacteriophage proliferation and electron microscopy

Tissue samples were subjected to phage detection by PCR (data unpublished). PCR-positive tissue samples were ground with normal saline, and PCR-positive bone infusions were supplemented with normal saline. The samples and infusions were then centrifuged at 1,500 rpm for 3 min and passed through a 0.45-µm syringe filter followed by a 0.22-µm syringe filter to ensure the removal of *Y. pestis*. The final filtrate (phage original cultures solution) was used for phage proliferation.

Single colonies of *Y. pestis* EV76 were inoculated into 5 mL of *Brucella* broth and cultured overnight. The resulting bacterial culture was transferred into 500 mL of *Brucella* broth for further cultivation. When the bacterial solution became slightly opaque and turbid, 10 mL of phage original cultures solution with a titer of 10^8^ PFU/mL was added and the culture was continued until the liquid was clear. The solution was centrifuged at 5,000 rpm for 10 min at 4°C. The supernatant was passed through a sterile 0.22-µm syringe filter to obtain crude bacteriophage lysates.

Crude bacteriophage lysates were filter sterilized using a 0.22-µm membrane (Millipore, Waltham, MA, USA) and then pelleted at 25,000 × *g* for 1 h at 4°C using a Beckman high-speed centrifuge (Beckman Coulter, Palo Alto, CA, USA). The phage pellet was washed two times in 0.1 M ammonium acetate and then re-suspended in 150 µL of SM buffer supplemented with CaCl_2_ (5 mM). Phage particles were deposited onto a carbon-coated Formvar membrane on copper grids, and stained with 20 µL of 2% potassium phosphotungstate (pH 7.2). After dye removal with filter paper, phage particles were examined under a transmission electron microscope (TECNAI 12; FEI, Hillsboro, OR, USA), operating at 120 keV. Images were collected and analyzed using Digital Micrograph Software (Gatan, Pleasanton, CA, USA). Taxonomic assignments were made according to the classification scheme for phages developed by Ackermann and Berthiaume and the International Committee on the Taxonomy of Viruses.

### Genome sequencing and assembly

DNA of *Y. pestis* AKS2022HT87 and 80 isolates of *Y. pestis* from the *M. himalayana* plague focus of the Qinghai–Tibet Plateau were extracted using the Wizard Genomic DNA Purification Kit (Promega, USA). A 10K SMRT Bell library was generated utilizing the SMRT Bell TM Template kit (version 2.0). The complete genome of *Y. pestis* was sequenced by Pacbio sequel, and the generated reads were assembled using Canu software (http://github.com/marbl/canu/, version 2.0). Phage sequences on the sample genomes were predicted by phiSpy software (version 2.3) (49).

Phage genomic DNA was extracted by conventional sodium dodecyl sulfate lysis and the phenol-chloroform method. The DNA samples were randomly sheared into fragments of approximately 350 bp using the Covaris ultrasonic disruptor. The fragmented DNA was then processed using the NEBNext Ultra DNA Library Prep Kit for Illumina (NEB, SA) to generate a complete DNA library. Phage genomes were sequenced using Illumina technology, and the phages were assembled according to the De Bruijn algorithm using the software packages SPAdes and SOAPdenovo. Average nucleotide identity (ANI) was determined among all pairwise combinations of phage genomes and was performed by R version 4.2.2. The putative open reading frame was predicted by Prokka.

To study the phylogenetic relationship and compare the genetic characteristics of *Y. pestis*. A phylogenetic tree was constructed using the neighbor-joining method based on 3,850 core genes from *Y. pestis* AKS2022HT87 and 80 isolates of *Y. pestis*. The genetic distance was calculated using Poisson correction and the unit is the number of amino acid substitutions per site. All positions containing gaps and missing data were eliminated. Visualization of the phylogenetic tree was performed using iTOL (50). Genome component prediction included the prediction of the coding gene, repetitive sequences, non-coding RNA, genomics islands, transposon, prophage, and clustered regularly interspaced short palindromic repeat sequences (CRISPR). *Y. pestis* genes used three databases to predict gene functions. They were respective COG (Clusters of Orthologous Groups), NR (Non-Redundant Protein Database databases), and Swiss-Prot. A whole genome Blast search (*E*-value less than 1e−5, minimal alignment length percentage larger than 40%) was performed against the above three databases.

### Statistical analyses

Statistical analyses were conducted using SPSS 24.0 (IBM Corp., USA). The non-normal distribution of the spleen-somatic index was described by median and IQR. The Kruskal-Wallis test was used to examine the differences in spleen-somatic index among groups (different marmot infection groups and months of sample collection). Dunnett’s test was used for pairwise comparison. Bilateral *P* < 0.05 was considered statistically significant.

## Data Availability

The genome sequence in this study is available in GenBank. The sequence of phage AKS2022HT87GU_phi was deposited in GenBank under accession number OR671250. The sequences of *Yersinia pestis* AKS2022HT87 were deposited in GenBank with accession numbers CP155632-CP155635 (strain A) and CP155628-CP155631 (strain B).
